# Chicago Medical Society

**Published:** 1881-06

**Authors:** 


ARTICLE V.

CHICAGO MEDICAL SOCIETY.

Stated meeting, May 2. Dr. E. Ingals, President, in the chair.

Dr. G. W. Earle read the paper of the evening on

ROETHELN, OR GERMAN MEASLES.

of which the following is an abstract:

"During the months of March and April we have been having a disease characterized by mild coryza, redness and suffusion of eyes, very slight fever, enlargement of the glands of the neck, followed with a papular eruption. These red spots are smaller than in measles and larger than in scarlet fever, and are entirely unlike variolous diseases. It is contagious, and in all its main features corresponds to what the Germans call Rötheln." Follows the history of twelve typical cases.

"Probably this disease is the one described as early as 1492 by Ali Abbas of Venice. As far back as the middle of the last century the German physicians wrote of an acute exanthem closely resembling this disease, and called it Rubeola. About that time the French and the English doctors called this disease Roseola. About half the authors and writers believe the disease under consideration to be either mild scarlatina, or measles, or both; and the other half believe it to be a separate affection. If this be an independent disease, as I believe it is, the first thing we want is a name.

"We should not call it Rötheln, because this is the name applied to Rubeola, and Rubeola is the name applied by many to measles. It is not roseola, for that is not contagious (the disease I am speaking about is certainly mildly contagious). It is manifestly improper to designate it German Measles, for this new progeny may first have seen the light on French or English soil.

ETIOLOGY.

"That this disease is both contagious and epidemic, I have no doubt. What is the contagious principle? We must say as we do in regard to scarlatina and measles, that we know nothing *per se* of this element. Of its predisposing causes we may be able to say something.

"*Its Relation to Miasmata.*—One observer has remarked that after great moisture, when the air is charged with impurities, the disease seems to prevail. The peculiar winter, from which we have emerged, may have had something to do as a cause of our present epidemic.

"*Incubation.*—This is from two and one-half to three weeks, according to Emminghaus. According to my experience, it is, with considerable regularity, seventeen days.

"*Age.*—More adults seem to be affected during our present epidemic than in those described by former writers. Infancy seems to be nearly exempt.

"*Social Position.*—The rich and poor alike seem to be liable to it.

SYMPTOMS AND HISTORY.

"Among children I have not been able to notice any prodromal period. In adults it is frequently present, and consists of languor, chilliness, pain in the muscles, surface of body feels flushed to patient, and in one of the cases a dizzy sensation has been experienced. In children, the first thing noticed will be the eruption, although in a few the eyes are somewhat red, and sneezing may precede the eruption. The coryza is not at all severe, and the conjunctival trouble has not in any of my cases except one amounted to photophobia.

"The pharynx is frequently red, not intensely so, and sometimes the redness is not diffused. The glands of the neck are swollen in about all the cases. The tonsils in one adult were considerably enlarged, and in one case a gland (the post auricular) was very largely swollen for days. The exanthem may be preceded a few hours by a redness of the forehead and cheeks—really an erythema—and then the spots make their appearance. I have noticed the eruption at first on the neck or chest, from which it spreads to other parts of the body.

"*Temperature.*—In the majority of cases it is normal. In a few cases, and especially in adults, when the pain in the head and pharynx is great, it is slightly increased. In one case it attained 104.

"*Pulse.*—The pulse in a patient seven years old, counted while I am dictating these words, is 90. Indeed, the symptoms in children are so mild that they hardly ever take to their beds, and some boys do not discontinue their play out-doors.

"*Complications.*—Pneumonia has taken place in three or four cases in 120. Aphthæ has followed in a few cases, and rheumatism in two or three cases. It was very slight. There has been a tendency to gastro-intestinal irritation in a few cases. Pain in the bowels continued for several days in one patient. I think there is a decided tendency to urticaria, and in one or two cases a pemphigus has been noticed, which, however, remained only a few hours. Slight malarial troubles may, and frequently do complicate the disease, especially in this climate. I have noticed a slight furfuraceous detachment in a few cases, especially about the nose. There is no desquamation. Two *relapses* have occurred in my practice—one at the end of the third day, the other after twenty-one days. The relapse in this case was attended with considerable pain.

DIAGNOSIS.

"This is the most important question in regard to the whole subject. It is liable to be confounded with variola or varioloid, scarlatina, erythema, urticaria, rubeola, and roseola. Not a few cases have in this epidemic been taken for the erythematous or papular stage which sometimes is seen in *small-pox.* The want of severe head and back-ache, and the rapid disappearance of the eruption in a few hours, dispel any doubts of this character. The want of sore throat, the mild fever, the papular eruption instead of a rash differentiates it from *scarlatina.* In *measles* a severe bronchitis is invariably present for a few days, and there is a rise in the temperature from 102° to 104°; the eruption is about three days in spreading over the body, and when fully developed is much larger than in the disease we are considering. The coryza and photophobia are severe in measles; they are mild in this disease, whose incubation is from seventeen to twenty days, while it is nine days in measles. An erythematous blush is present, usually confined to a small area, as the forehead or one cheek. *Roseola*, more than any other disease, will give trouble in diagnosis. Roseola is said to be sometimes epidemic, but no one has claimed for it the element of contagion. Rötheln has a period of incubation, and is markedly contagious. Roseola has a prodromal period. Rötheln has but few, if any, premonitory symptoms.

RECAPITULATION.

We have, then, a disease occupying about the same relation to measles and scarlatina that varicella does to the variolous diseases. It is a specific, acute exanthem, beginning by usually slight coryza and suffusion of the eyes, followed by a papular eruption, enlargement of cervical glands. The *treatment* should be conducted on general principles."

DISCUSSION.

Dr. F. M. Weller had seen some severe cases of this disease in Evanston in 1873. A few of them died. Dr. J. S. Jewell had seen the cases in consultation. A feature of his treatment was to expose a mixture of two parts of carbolic acid with one of camphor in a dish on the floor of the sick-room. He claimed this prevented the disease spreading to other members of the family.

Dr. G. C. Paoli said that, although he had seen several hundred cases of rötheln, he had never known one to die of that disease, and that cases of death could always be referred to scarlatina or some other disease present in the same locality. This disease had been described by Arabian authors long ago, but it was long before it was differentiated from scarlatina. A peculiarity of rötheln is that the eruption always commences on the face or forehead. The absence of of albuminuria would help the diagnosis in cases where scarlatina might be present.

Dr. Roswell Park is just now engaged in the management of more than a hundred cases in the Orphan Asylum, where the disease has occurred as an epidemic. An epidemic of measles had happened in the same institution a year ago. His observations agreed with those of Dr. Earle. But he had noticed that the eruption sometimes appeared first at the roof of the mouth. There was a mild sore throat in most of the cases; twenty per cent. had bronchitis; several had photophobia. He had not seen any cases of intestinal trouble. A young woman affected with rötheln had complained of pain in the legs and back. Perhaps it depended on malaria. The spots of the eruption were a millimeter in diameter. Three or four cases exposed to cold had had pneumonia or severe bronchitis. He had not met any deaths. His treatment consisted of aconite, bromides to relieve the headache, laxatives when required, and co. syrup of squills to relieve the bronchitis. Most of his cases began as a bronchial trouble. The Society adjourned. H. D. V.

